# Genetic diversity, multiplicity of infection and population structure of *Schistosoma mansoni* isolates from human hosts in Ethiopia

**DOI:** 10.1186/s12863-015-0297-6

**Published:** 2015-12-03

**Authors:** Mulugeta Aemero, Jérôme Boissier, Deborah Climent, Hélène Moné, Gabriel Mouahid, Nega Berhe, Berhanu Erko

**Affiliations:** Microbial, Cellular and Molecular Biology Program Unit, College of Natural Science, Addis Ababa University, P. O. Box 1176, Addis Ababa, Ethiopia; University Perpignan Via Domitia, IHPE UMR 5244, CNRS, IFREMER, University Montpellier, F-66860 Perpignan, France; Aklilu Lemma Institute of Pathobiology, Addis Ababa University, P. O. Box 1176, Addis Ababa, Ethiopia; Department of Biology, College of Natural and Computational Sciences, University of Gondar, P. O. Box 196, Gondar, Ethiopia

**Keywords:** *Schistosoma mansoni*, Microsatellite, Genetic diversity, Population structure, Ethiopia

## Abstract

**Background:**

Human intestinal schistosomiasis caused by *Schistosoma mansoni* and urinary schistosomiasis caused by *Schistosoma haematobium* are endemic in Ethiopia. Although schistosomes look morphologically uniform, there is variation in infectivity, egg productivity and virulence due to variation in their genetic make. Knowing the genetic diversity and population structure of *S. mansoni* isolates will enable to understand and consider the possible variability in terms of infectivity, egg productivity and virulence.

**Methods:**

Between 2010 and 2011, genetic diversity and population structure of *Schistosoma mansoni* isolates from four endemic areas of Ethiopia was assessed using previously published 11 polymorphic microsatellite loci. Miracidia were hatched from eggs of *S. mansoni* collected from stools of human subjects residing in Kemissie, Wondo Genet, Ziway and Sille-Elgo villages. DNA was extracted from single miracidium and PCR was run following standard protocol. Allelic polymorphism and population genetic structure was analyzed using different software.

**Result:**

At a population level (i.e. different villages), the mean number of alleles per locus, allelic richness, expected heterozygosity in Hardy–Weinberg equilibrium and pairwise *F*_*ST*_ values ranged from 8.5 to 11.5, 3.46–20.8, 0.66–0.73 and 3.57–13.63 %, respectively. All analyzes on population genetic structure reveals strong genetic structuration corresponding to the four sampled villages. At infrapopulation level (i.e. different hosts) the mean number of alleles per locus, allelic richness, expected heterozygosity in Hardy–Weinberg equilibrium and *F*_*IS*_ values ranged from 3.09 to 7.55, 1–1.96, 0.59–0.73 and 0.1763–0.4989, respectively. Mean estimated genetically unique adult worm pairs within hosts ranged from 66 to 92 % revealing the occurrence of infection of a single host with genetically unique multiple *S. mansoni* strains. The data also indicated the occurrence of genetic variation within inter- and intra-hosts.

**Conclusion:**

High level of genetic diversity and significant population differentiation characterized the *S. mansoni* isolates of Ethiopia. These results are quite different from previous studies demonstrating that it is difficult to generalize schistosome transmission patterns because epidemiological situation tends to vary. These are important factors to be considered in relation with morbidity, drug resistance or vaccine development.

## Background

Human intestinal schistosomiasis caused by *Schistosoma mansoni* is endemic in Ethiopia. Varying in distribution and magnitude of disease burden, *S. mansoni* is widely distributed throughout the country and hence is a major public health problem [1–3].

Schistosomes reproduce sexually reproduction in their definitive hosts (humans, other primates, rodents) and this type of reproduction allows for reassortment and the perpetuation of parasite genotypic diversity. The eggs laid by the adult female worms pass in the host’s faeces and each will hatch in water to release a miracidium. These free-swimming larvae must find and penetrate an appropriate freshwater snail, in the case of *S. mansoni*, a snail of the genus *Biomphalaria*. Once it has penetrated the snail, the miracidium transforms into a mother sporocyst which produces multiple daughter sporocysts through asexual reproduction. These in turn produce cercariae which are released into water and are infective to human hosts, thus completing the life cycle.

Although *S. mansoni* is considered morphologically uniform, it is known that strains from the same or different geographical locations have shown differences in egg production, infectivity, pathogenicity and susceptibility to chemotherapy [4]. These characteristics could be due to difference in the population genetic structure of schistosomes. A previous study had indicated the presence of genetic diversity in *S. mansoni* parasites [5]. Study on genetic composition of natural populations of the parasite *S. mansoni* in northern Senegal using nine microsatellite markers revealed a random distribution (panmixia) of parasite genetic variation among villages and hosts, confirming the concept of human hosts as ‘genetic mixing bowls’ for schistosomes [6]. Though host sex and village of residence did not show any association with parasite genetics, host age was significantly correlated with parasite inbreeding and heterozygosity, with children being more infected by related parasites than adults. The study suggests that host-specific factors, such as age and concomitant immunity, may shape the genetic composition of schistosome populations, revealing important insights into host–parasite interactions within a natural system [6–8]. Thus elucidating the distribution of parasite genetic diversity is critical to the understanding and prediction of disease epidemiology. One of the primary reasons for studying parasite population genetics is to understand demographic parameters, such as gene flow and population size, which are not readily observable using conventional ecological methods. These insights allow inferences regarding the patterns of parasite transmission and recruitment within the environment [9]. Moreover, by using molecular tools, studying the population genetic structure will enable to determine whether changes in gene frequencies provide insight into the effectiveness of treatment, understand the impacts of treatment on the gene pool and population structure of *Schistosoma* parasites, and establish whether movement of humans from refugia or non-treated areas introduces new parasites into local populations [10, 11]. A meta-analysis of eight *Schistosoma mansoni* (two published and six unpublished) microsatellite datasets collected from individual schistosome-infected school-children showed that *S. mansoni* populations were more diverse in East than West African schools, but heterozygosity levels did not vary significantly with geography [12]. Genetic structuring was also detected in *Schistosoma mansoni* and *Schistosoma haematobium* populations from different countries in sub-Saharan Africa, indicative of isolation by distance [12]. In other studies of African countries there is a link between schistosome infection intensity, transmission and parasite genotype and the genetic structure of worm populations [5]. Although considerable epidemiological studies have been conducted in Ethiopia, no work has been done on molecular characterization of *S. mansoni* so far. Our previous epidemiological survey performed on three villages from northern, central and southern Ethiopia shows considerable variation in terms of prevalence and intensities of infections [1]. The prevalence of *Schistosoma mansoni* infection among the study participants in Kemissie, Wondo Genet and Sille-Elgo was 89.6, 59.9, and 31.6 %, respectively. The highest and geometric mean of egg per gram of stool for Kemissie, Wondo Genet and Sille-Elgo was, 5208 and 346, 8472 and 252, 3960 and 91, respectively [1]. Therefore, due to different epidemiological patterns and infection intensities of *S. mansoni* observed across these villages, in this present study it was hypothesised that there will be substantial difference in genetic diversity between isolates of Ethiopian *S. mansoni* populations.

## Methods

### Study area

The study was conducted in four geographically distant *Schistosoma mansoni* endemic areas namely: Wondo Genet about 261Km south of Addis Ababa, located at 07°05′35″N, 038°36′66″E at an altitude of 1755 m above sea level, Ziway about 164Km away from the capital Addis Ababa, located at 07°56′37″N, 038°43′25″E at an altitude of 1642 m above sea level, Sille-Elgo about 525Km away southwest of Addis Ababa located at about 05°28′39″N, 037°26′02″E at an altitude of 1188 m above sea level, and Kemissie about 305 km northeast of Addis Ababa located at 10°43′30″N, 039°04′20″E at an altitude of 1450 m above sea level. Work permission was obtained from local administrative officers, health offices and school principals.

### Study design

From 2010 to 2011 a cross sectional parasitological study was conducted in the four study areas in order to determine the prevalence and intensity of *Schistosoma mansoni* infection. Among those found with high intensity of S*chistosoma mansoni* infection, stool specimens were collected for the second time to harvest egg and hatch miracidia. Each single miracidium was used to determine genetic diversity and population structure of *Schistosoma mansoni* isolates from the four endemic study areas.

### Stool collection, examination, and miracidia hatching

Small plastic sheets were distributed to voluntary study participants and sizable stool specimens were collected and examined using Kato-Katz method (41.7 mg template) [13]. Infection status was determined by the presence or absence of *Schistosoma mansoni* eggs. Stool specimens were collected from 16 *S. mansoni* positive subjects in Sille-Elgo, 30 subjects in Wondo Genet, 30 subjects in Kemissie and15 subjects from Ziway, totaling 91. The stool specimens were kept in 0.85 % saline in vial and transported in ice box to the Medical Parasitology Laboratory of Aklilu Lemma Institute of Pathobiology, Addis Ababa University, to harvest miracidia.

In order to stimulate hatching of miracidia, stool samples were homogenized with saline and sieved through tiered sieve of 425, 180 and 140 μm mesh size and kept for about 20 min in dark in order to allow the eggs to settle in the bottom of the flask. The supernatant was poured and the eggs were put in 250 ml flask filled with aged water. The flasks were exposed to artificial light in order to initiate hatching. The flasks were covered with black carbon paper and aluminum foil. This induces the positive phototropic and negative geotropic characteristics of the miracidia which results in their accumulation on the top of the flask [14]. From those specimens that hatched a total of 379 miracidia from 52 patients were collected. These included 81 miracidia from seven individuals in Sille-Elgo, 88 miracidia from 19 individuals in Wondo Genet, 151 from 20 individuals of Kemissie, and 59 from six individuals in Ziway. The miracidia were transferred individually in 2 μl of water using micropipette into Eppendorf tube under a dissecting microscope. Single miracidium was put in Eppendorf tube in 96 % ethanol at −20 °C until processed in the laboratory of Centre de Biologie et d’Ecologie Tropicale et Méditerranéenne, University of Perpignan Via Domitia, France.

### DNA extraction

DNA extraction from *Schistosoma mansoni* miracidia was done following Beltran et al. [15] protocol. In brief, before DNA extraction, miracidia were individually vacuum-dried for 15 min in a Speedvac evaporator. Following, 20 μl of NaOH (250 mM) was added to each tube. After a 15 min incubation period at 25 °C, the tubes were heated in boiling water at 99 °C for 2 min. Then, 10 μl HCl (250 mM), 5 μl of Tris–HCl (500 mM) and 5 μl Triton X-100 (2 %) were added and a second heat shock in boiling water at 99 °C for 2 min was performed. The products were put in room temperature until processed for Polymerase Chain Reaction (PCR).

### Polymerase chain reaction

Previously published 11 polymorphic microsatellite markers, namely SMDA28, SMC1, SMDO11 and AF325698 [16], R95529, SMD57, L46951 and SMD25 [17], SMBR16 and SMBR10 [18] and SMS7 [19] were used to determine the genetic variation and *Schistosoma mansoni* population structure of the Ethiopian isolates. To maximize efficiency and minimize cost, these PCRs were performed in three multiplexes [20]. The PCR amplifications loci: R95529, SMC1, SMBR16, SMD57, SMDO11 are Multiplex1; SMDA28, SMS7, SMD28 are Multiplex2, and SMBR10, L46951, SMD25 are Multiplex 3. The PCR reactions were carried out in a total volume of 20 μl containing 4 μl of 5X buffer (10 mM Tris–HCl, pH 9.0 at 25 °C, 50 mM KCl, 0.1 % Triton X-100), 0.2 μM of each oligonucleotide primer, 200 μM of each dNTP (Promega), 1 unit of GoTaq polymerase (Promega, Madison, Wisconsin), 1 μl of extracted DNA and DNase-free water q.s.p. 20 μl. The PCR program consisted an initial denaturation phase at 95 °C for 5 min, followed by 40 cycles at 95 °C for 30 s, 57 °C annealing temperature for 20 s, 72 °C for 30 s, and a final extension at 72 °C for 10 min in a thermocycler (Bio-Rad, Hercules, USA). For each marker, the forward PCR primer was 5̍ fluorescein labeled (Proligo, Cambridge, UK) allowing a precise analysis in an automated DNA sequencer. A mix of 40 μl sample loading solution (Beckman Coulter, Villepinte, France) and 0.1875 μl DNA size 400, a red labeled size standard (CEQTM DNA size standard kit, 400 Beckman Coulter), was prepared and 0.75 μl of the microsatellite PCR products were diluted in 39.25 μl sample loading solution. Mineral oil was dropped in each tube and electrophoresed using an automatic sequencer (CEQTM 8000, Beckman Coulter) with CEQTM 8000 sequence analysis software. The sizes of the alleles were calculated using the fragment analyzer package [20]. All loci were tested pairwise based on 4400 permutations and adjusting *P* value to 0.000227 and there was no linkage disequilibrium detected.

### Data analysis

In this study, miracidia from all the patients within a single study site were treated as a population while all miracidia in a single host are treated as infrapopulation.

The allele frequencies were calculated using the program MICROSATELLITE TOOLKIT (software available upon request). Both the expected and observed heterozygosities were also calculated using MICROSATELLITE TOOLKIT and their statistical significance tested using the chi-square test at α̍ =0.05. FSTAT was used to test for deviations from Hardy-Weinberg equilibrium using exact tests, testing the hypothesis that observed diploid genotypes are the product of random union of gametes. An exact test for linkage disequilibrium between pairs of loci was performed using the FSTAT. Mean estimates of *F*_*IS*_ (inbreeding coefficient) for each population and pairwise *F*_*ST*_ (between all population pairs) were also calculated following the method of Weir and Cockerham [21]. Deviation of *F*_*IS*_ and *F*_*ST*_ values from zero was tested using a permutation test. All *F* statistics were carried out using FSTAT 2.9.3.2 [22]. Isolation By Distance (IBD) was tested correlating genetic distance (Fst/(1-Fst)) and geographic distance in kilometer. The paired *t*-test is used to compare two sample means where there is a one-to-one correspondence (or pairing) between the samples while Friedman’s test was used for ordinal data or an interval-scale variable that is not normally distributed [23]. Genetic structuration was assessed using both Principal Component Analysis (PCA) using Genetix software [24] and bayesian approach using Structure software [25]. Full-sib analyses was assessed by estimating mean number of genetically unique adult worm pairs and its standard deviation for each patient using Colony software [26].

### Ethical consideration

The study was ethically approved by the Institutional Research and Ethics Committee of the Department of Microbial, Cellular and Molecular biology, Addis Ababa University, P.O. Box 1176, Addis Ababa, Ethiopia and by the National Research Ethics Review Committee of Federal Republic of Ethiopia Ministry of Science and Technology, P.O. Box 2490, Addis Ababa, Ethiopia. Informed verbal consent was obtained from all adults. For school age children younger than 18, informed verbal consent was obtained from their parents through health extension workers and school principals. In addition, the children also gave their assent. All study participants found positive for *S. mansoni* were treated with Praziquantel at a dose of 40 mg/Kg body weight.

## Results

### Population level

Out of the 379 miracidia collected from 41 *Schistosoma mansoni* positive individuals (nine from Kemissie, 19 from Wondo Genet, seven from Sille-Elgo and six from Ziway) a total of 288 were successfully genotyped for 11 loci and analyzed at population level.

One hundred sixty four alleles were scored in all of the four populations for all of the 11 loci examined. There was no null allele detected. Individually a total of 127, 123, 102 and 94 alleles for all of the 11 loci were counted for Kemissie, Wondo Genet, Sille-Elgo and Ziway, respectively. The number of alleles scored for each locus ranged from 4 to 22 (SMC1-SMDO11) with a mean value of 8.5 in Ziway; from 3 to 25 (SMD28-SMDO11) with a mean value of 11.5 in Kemissie; 4–22 (SMC1-SMDO11) with a mean value of 11.2 in Wondo Genet and, 3–23 (SMD28-SMDO11) with a mean value of 9.3 in Sille-Elgo for the 11 loci. The number of alleles in all of the four populations ranged from 8 to 34 (SMD28-SMDO11) with a mean value of 14.9 (Table 1). Nonparametric Friedman test showed significant difference in number of alleles counted within the populations (*χ*^2^ = 10.941 at 3DF; *P* = 0.012).

**Table 1:** Number of allele count (A) in relation to each locus of microsatellite in the four study populations

Locus	Kemissie	Sille-Elgo	Wondo Genet	Ziway	All populations
R95529	11	4	11	5	14
SMC1	5	7	4	4	7
SMBR16	11	10	10	9	14
SMD57	17	15	19	11	21
SMDO11	25	23	22	22	34
SMDA28	14	7	12	11	15
SMS7	4	4	5	6	8
SMD28	3	3	6	4	6
SMBR10	12	11	11	9	15
L46951	13	9	12	9	16
SMD25	12	9	11	4	14
Mean	11.5	9.3	11.2	8.5	14.9

Allelic richness was calculated based on minimum sample size of 29 diploid individuals (Table 2). It ranged from 3.46 to 20.8 (SMD28-SMDO11) with a mean value of 9.9 in all of the four populations. Ziway had allelic richness value of 3.6–22.28 (SMD28-SMDO11) with a mean value of 9.06, Kemissie 2.99–18.93 (SMD28 -SMDO11) with a mean value of 9.44, Wondo Genet 3.79–16.81(SMD28-SMDO11) with a mean value of 9.18 and Sille-Elgo 3.0–18.16 (SMD28-SMDO11) with a mean value of 7.71. The nonparametric Friedman test showed no significant difference in allelic richness among the four populations (*χ*^2^ = 3.327 at 3DF; *P* = 0.344). However, Wilcoxon rank test indicated that Sille-Elgo had the lowest allelic richness with significant deference from both Kemissie and Wondo Genet (*P* < 0.05).

**Table 2:** Allelic richness (An) in relation to each locus of microsatellite in the four study populations (minimum sample size of 29 diploid individuals)

Locus	Kemissie	Sille-Elgo	Wondo Genet	Ziway	All populations
R95529	9.20	4.00	8.31	4.53	8.01
SMC1	4.68	6.38	3.99	4.00	5.22
SMBR16	9.82	8.35	8.52	9.00	11
SMD57	14.20	12.95	15.19	10.53	15.7
SMDO11	18.93	18.16	16.81	18.86	20.8
SMDA28	10.40	5.90	10.71	10.87	10.75
SMS7	3.52	3.66	4.25	5.75	4.64
SMD28	2.99	3.00	3.79	3.60	3.46
SMBR10	10.04	7.42	9.90	8.42	9.92
L46951	9.77	7.35	10.09	8.08	10.1
SMD25	10.28	7.62	9.40	3.99	9.3
Mean	9.44	7.71	9.18	7.97	9.9

Paired *t*-test was used to evaluate the statistical significance of the deviations between the observed and expected heterozygosity (*t*(10.367), 43DF, *P* = 0.000) (Table 3). The expected heterozygosity was 73 % in Sille-Elgo followed by Wondo Genet (71 %), Kemissie (69 %) and Ziway (66 %). Similarly the observed heterozygosity was 52 % for Sille-Elgo 49 % for Wondo Genet, 44 % for Kemissie and 37 % for Ziway. In all of the study populations *F*_*IS*_ has a high positive value different from zero for all of the 11 loci while only Sille-Elgo and Ziway had a respective negative value at the loci SMDA28 and SMBR10 (Table 4). The mean *F*_*IS*_ value for all of the populations ranged from 0.27853 to 0.4347. Statistical test showed that there is high significant difference between the expected and observed heterozygosities (*χ*^2^ = 32.818, 1DF, *P* = 0.000).

**Table 3:** Total number of sample size, loci typed and expected (H_E_) and observed heterozygote (H_O_) of the four study populations

Population	Sample size	Loci typed	*H*_*E*_	*H*_*E*_ Standard deviation	*H*_*O*_	*H*_*O*_ Standard deviation
Kemissie	79	11	0.6975	0.0686	0.4427	0.0187
Sille-Elgo	79	11	0.7308	0.0352	0.5283	0.0180
Wondo Genet	82	11	0.7180	0.0657	0.4954	0.0181
Ziway	48	11	0.6668	0.0634	0.3771	0.0231

**Table 4:** Expected (H_E_) and observed heterozygote (H_O_) of each population in relation to each locus with F_*IS*_ value

	Kemissie		Sille-Elgo		Wondo Genet		Ziway		Kemissie	Sille-Elgo	Wondo Genet	Ziway
Locus	H_E_	H_O_	H_E_	H_O_	H_E_	H_O_	H_E_	H_O_	*F*_*IS*_	*F*_*IS*_	*F*_*IS*_	*F*_*IS*_
R95529	0.81	0.34	0.59	0.40	0.72	0.50	0.29	0.16	0.58564	0.32500	0.30795	0.451
SMC1	0.69	0.42	0.72	0.67	0.64	0.48	0.57	0.34	0.39290	0.07477	0.24957	0.40111
SMBR16	0.78	0.43	0.81	0.58	0.79	0.48	0.84	0.41	0.44630	0.28459	0.38707	0.51092
SMD57	0.89	0.42	0.89	0.65	0.90	0.43	0.86	0.38	0.53463	0.27434	0.52226	0.53747
SMDO11	0.91	0.75	0.92	0.56	0.83	0.60	0.91	0.67	0.17017	0.38913	0.27443	0.27204
SMDA28	0.79	0.44	0.61	0.71	0.86	0.52	0.87	0.19	0.43963	−0.15352	0.39762	0.78089
SMS7	0.29	0.18	0.60	0.41	0.60	0.55	0.66	0.42	0.39753	0.30713	0.08779	0.36426
SMD28	0.24	0.16	0.66	0.40	0.12	0.10	0.35	0.22	0.35285	0.39274	0.19567	0.37233
SMBR10	0.75	0.64	0.66	0.62	0.81	0.66	0.56	0.57	0.15017	0.0698	0.18587	−0.02768
L46951	0.66	0.49	0.77	0.47	0.78	0.44	0.73	0.43	0.24916	0.39094	0.43511	0.40733
SMD25	0.86	0.60	0.80	0.34	0.86	0.69	0.70	0.35	0.30692	0.57500	0.19953	0.50472
Mean	0.70	0.44	0.73	0.53	0.72	0.5	0.67	0.38	0.36708	0.27853	0.31163	0.43473

The *F*_*ST*_ values are all significant and range from 3.57 (between Kemissie and Wondo Genet) to 13.63 % (between Ziway and Kemissie) (Table 5). The geographic distance between Kemissie and Sille-Elgo is 542.17Km while between Ziway and Kemissie is 553.55Km. The respective geographic distance between Kemissie and Wondo Genet, Ziway and Wondo Genet, Ziway and Sille-Elgo and, Wondo Genet and Sille-Elgo is 422.8Km, 132.5Km, 127.3Km and 178Km. Also the genetic distance between Kemissie and Ziway is 16.21 %, Kemissie and Sille-Elgo is 10.7 %, Sille-Elgo and Wondo Genet is 10.1 %, Sille-Elgo and Ziway is 8.99 %, Wondo Genet and Ziway is 8.21 % and, Kemissie and Wondo Genet is 3.8 % (Table 6). No link between genetic distance and geographic distance (IBD) is observed (Spearman correlation *n* = 6, *r* = 0.6, *p* = 0.24).

**Table 5:** Population pairwise F_ST_ values for the study populations in relation to the 11 loci

Location	Ziway	Kemissie	Wondo genet	Sille-Elgo
Ziway	_	13.63 %	7.55 %	8.08 %
Kemissie	*	_	3.57 %	10.17 %
Wondo Genet	*	*	_	9.15 %
Sille-Elgo	*	*	*	_

(*F*_*ST*_ above diagonal and their significance level below diagonal, * *p* < 0.05)

**Table 6:** Matrices of genetic distance [Fst/(1-Fst)] (above diagonal) and geographic distance in Km (below diagonal) among and within study population areas

Population	Kemissie	Sille-Elgo	Wondo Genet	Ziway
Kemissie		10.75 %	3.8 %	16.21 %
Sille-Elgo	542.17		10.14 %	8.99 %
Wondo Genet	422.8	178		8.21 %
Ziway	553.55	127.3	132.5	

**Fig. 1:**
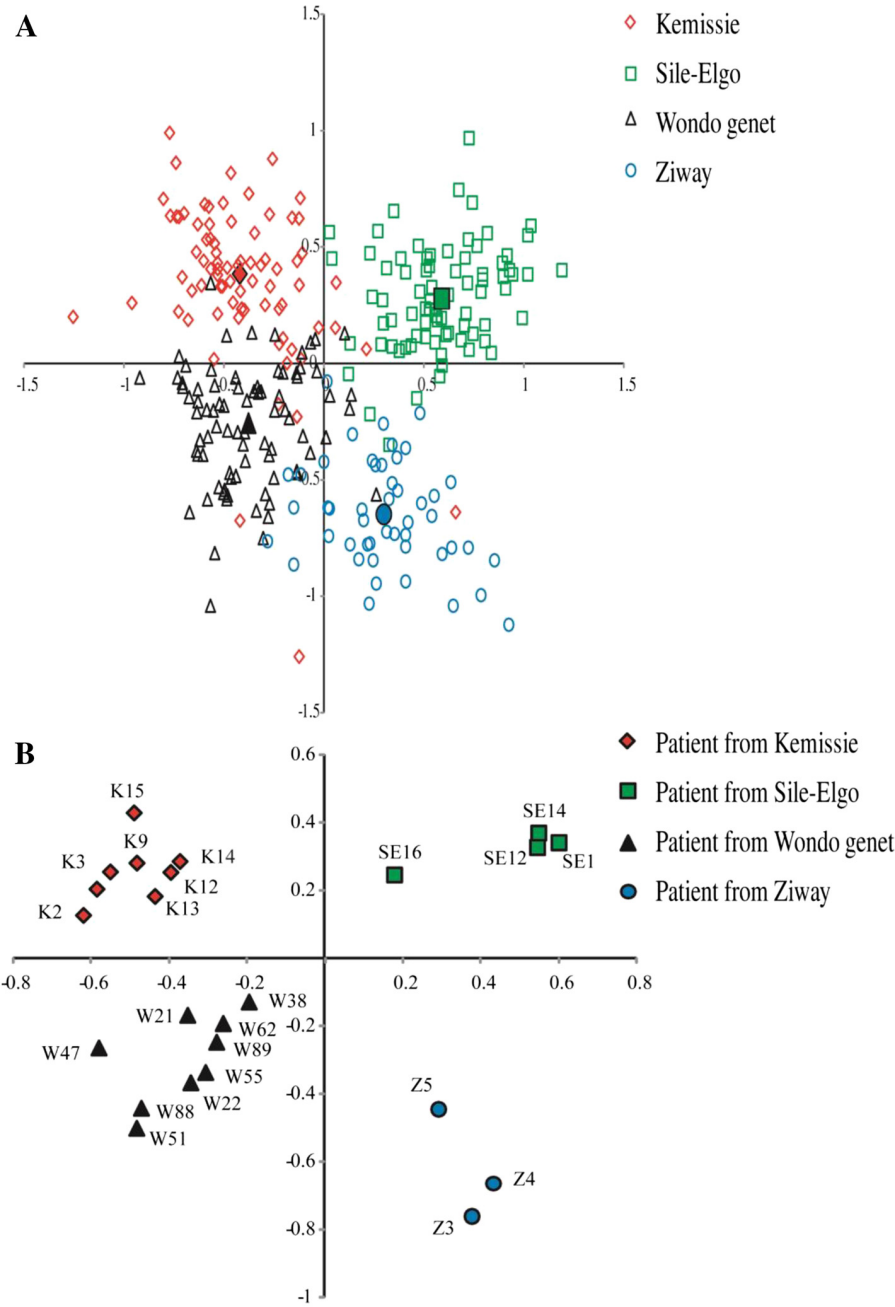
**a** Principal components analysis at population level. First two principal components (PCs) are shown here. Each miracidia is represented by one dot and the color label corresponding to their self-identified population origin (Kemissie, Sile-Elgo, Wondo Genet, Ziway). The percentage of the variation in genetic distances explained by each PC is 46.9 and 34.9 % for PC1 and PC2, respectively. **b** PCA by patient (each point represent one patient). The percentage of the variation in genetic distances explained by each PC is 16.9 and 11.5 % for PC1 and PC2, respectively

**Fig. 2:**
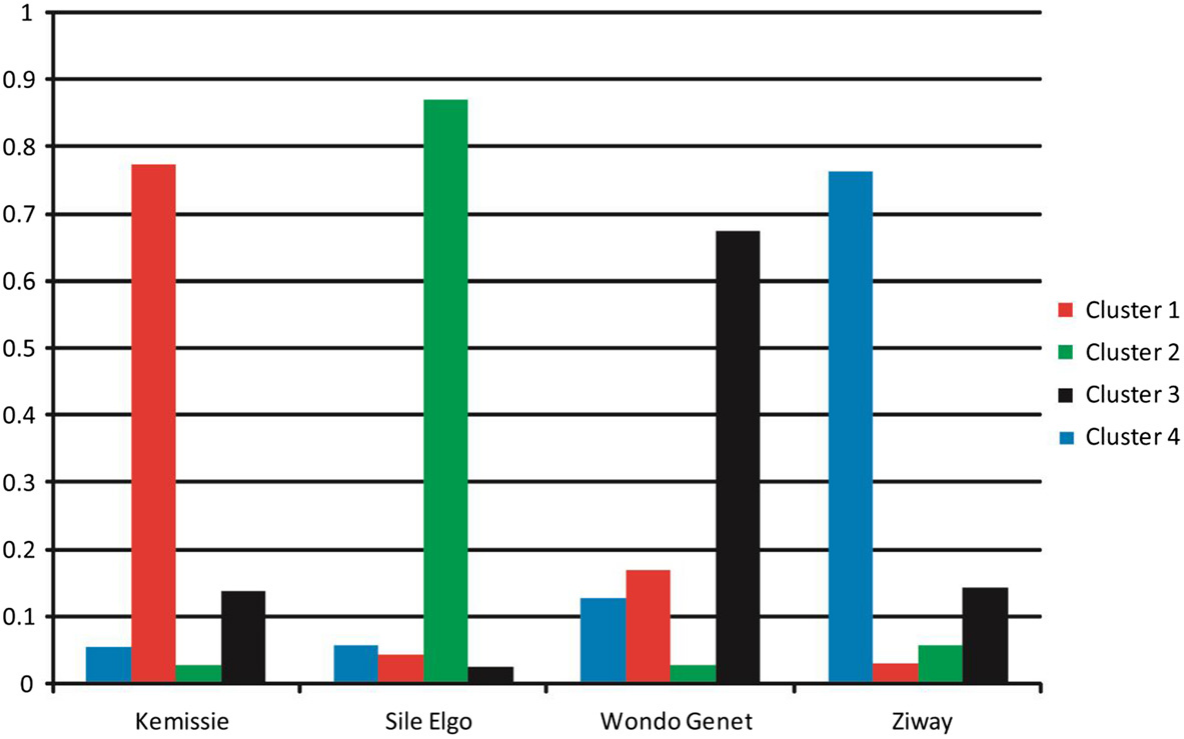
Cluster grouping inferred by Structure. The four colors correspond to the four clusters defined by Structure

Spatial genetic structuration or distribution of *Schistosoma mansoni* isolates in this study was determined by both Principal Component Analyses (PCA) using Genetix software [24] and Bayesian approach using Structure software [25]. The first two axes of the PCA (46.9 and 34.9 % of the total variation for principal component 1 and 2, respectively) split miracidia into four groups of points corresponding to the four villages (Fig. 1a). Only some miracidia from Kemissie are in the Wondo-Genet or Ziway group of points. Each patient could be affected to its own village (Fig. 1b). Similar result was obtain using the Bayesian approach, showing the maximum probability for four clusters (Fig. 2). Cluster 1 represents the structure of Ziway with 77 % of its own and sharing about 23 % from the rest. Cluster 2 represents Kemissie with 78 % of its own and 22 % sharing with the rest. Cluster 3 represents Sille-Elgo with 87 % of its own and sharing 13 % from the rest. Cluster 4 represents Wondo Genet having 68 % of its own and sharing 32 % with the others.

### Infrapopulation level

Twenty four infrapopulations were analyzed for the number of alleles counted. The scored number of alleles in individual study subjects which were represented by five or more miracidia also had shown variation (Table 7). The number of allele count at a single locus for the 24 infrapopulations range from 1 to 17. The largest mean number of allele was 7.54 and the lowest was 3.09.

**Table 7:** Number of allele count in relation to each microsatellite within a single individual human host

Locus	K1	K2	K3	K9	K12	K13	K14	K15	SE1	SE12	SE14	SE16	W21	W22	W38	W47	W51	W55	W62	W88	W89	Z3	Z4	Z5
R95529	6	4	5	8	4	4	4	7	3	4	4	3	2	4	4	5	3	3	5	3	4	2	4	2
SMC1	4	4	4	3	5	5	4	3	4	7	6	5	3	3	2	4	3	2	4	3	3	2	4	2
SMBR16	4	4	5	4	8	6	6	5	6	9	10	4	1	5	5	5	3	5	4	5	2	4	8	4
SMD57	7	6	6	8	10	10	8	6	5	12	12	5	7	7	6	5	6	6	4	7	6	3	10	3
SMDO11	7	12	7	9	14	9	7	4	7	16	14	5	5	5	9	8	3	3	7	5	5	5	17	8
SMDA28	6	9	5	5	7	8	7	4	4	6	5	3	2	5	7	5	5	5	6	3	10	5	10	4
SMS7	2	1	2	2	2	2	4	2	3	3	3	4	3	3	2	3	3	3	3	1	3	5	5	2
SMD28	2	2	1	3	2	2	2	2	3	3	3	3	1	1	3	1	1	2	1	1	1	2	4	1
SMBR10	7	7	6	5	7	5	7	5	3	7	8	5	4	6	6	5	5	5	5	5	4	5	9	2
L46951	6	7	6	3	7	5	4	3	6	5	9	4	3	4	4	5	5	4	4	3	5	4	8	3
SMD25	4	5	6	7	7	9	7	7	3	7	6	5	5	5	6	6	7	4	6	6	7	3	4	3
Total	55	61	53	57	73	65	60	48	47	79	80	46	36	48	54	52	44	42	49	42	50	40	83	34

Allelic richness of *Schistosoma mansoni* isolates at individual host level ranged from 1 to 1.96 for each locus (Table 8). For all of the 24 infrapopulations, the allelic richness for each locus was in the range of 1.42–1.93 with a mean value of 1.76. The lowest and highest allelic richness observed was at SMD28 and SMDO11 loci, respectively. There is no statistically significant variation in allelic richness of *Schistosoma mansoni* among each infrapopulations (*χ*^2^ = 12.023 at 23DF; *P* = 0.970).

**Table 8:** Allelic richness of infrapopulations based on minimum sample size of one diploid individual

	K1	K2	K3	K9	K12	K13	K14	K15	SE1	SE12	SE14	SE16	W21	W22	W38	W47	W51	W55	W62	W88	W89	Z3	Z4	Z5	All
R95529	1.88	1.58	1.89	1.88	1.73	1.73	1.64	1.80	1.28	1.56	1.64	1.61	1.43	1.71	1.65	1.80	1.71	1.46	1.82	1.73	1.82	1.20	1.26	1.53	1.70
SMC1	1.70	1.76	1.78	1.54	1.73	1.67	1.50	1.58	1.75	1.74	1.70	1.80	1.83	1.62	1.49	1.68	1.62	1.36	1.68	1.53	1.60	1.36	1.50	1.53	1.70
SMBR16	1.82	1.50	1.76	1.66	1.88	1.80	1.85	1.89	1.79	1.84	1.78	1.79	1.00	1.83	1.85	1.74	1.73	1.93	1.74	1.76	1.43	1.87	1.83	1.87	1.84
SMD57	1.89	1.84	1.86	1.87	1.87	1.93	1.85	1.78	1.82	1.89	1.89	1.84	1.93	1.96	1.91	1.83	1.89	1.91	1.71	1.91	1.88	1.71	1.84	1.73	1.92
SMDO11	1.89	1.94	1.81	1.92	1.95	1.91	1.90	1.80	1.88	1.93	1.91	1.93	1.79	1.89	1.94	1.94	1.71	1.71	1.93	1.73	1.82	1.80	1.92	1.96	1.93
SMDA28	1.82	1.89	1.81	1.55	1.52	1.70	1.81	1.76	1.50	1.66	1.59	1.64	1.54	1.83	1.91	1.83	1.85	1.82	1.80	1.68	1.93	1.89	1.86	1.87	1.84
SMS7	1.13	1.00	1.14	1.44	1.17	1.13	1.59	1.41	1.60	1.57	1.57	1.71	1.73	1.71	1.17	1.51	1.71	1.73	1.68	1.00	1.58	1.87	1.63	1.53	1.58
SMD28	1.11	1.11	1.00	1.49	1.21	1.47	1.11	1.47	1.57	1.64	1.67	1.60	1.00	1.00	1.44	1.00	1.00	1.20	1.00	1.00	1.00	1.20	1.41	1.00	1.42
SMBR10	1.82	1.78	1.79	1.66	1.58	1.82	1.83	1.79	1.51	1.67	1.67	1.76	1.75	1.78	1.87	1.87	1.67	1.76	1.87	1.80	1.64	1.80	1.53	1.43	1.72
L46951	1.68	1.74	1.77	1.43	1.76	1.62	1.55	1.62	1.82	1.77	1.78	1.82	1.69	1.65	1.79	1.67	1.83	1.71	1.80	1.60	1.80	1.78	1.73	1.71	1.83
SMD25	1.77	1.79	1.81	1.83	1.82	1.92	1.87	1.86	1.66	1.79	1.73	1.84	1.82	1.83	1.76	1.89	1.93	1.75	1.84	1.89	1.77	1.61	1.69	1.83	1.84
Mean	1.68	1.63	1.67	1.66	1.66	1.70	1.68	1.71	1.65	1.73	1.72	1.76	1.59	1.71	1.71	1.71	1.70	1.67	1.72	1.60	1.66	1.64	1.65	1.64	1.76

For the 24 infrapopulations a total of 247 miracidia were analyzed at 11 loci level for their heterozygosity (Table 9). It was observed that, the value is higher for the expected heterozygosity (59–73 %) than the observed heterozygosity (28–59 %). Paired *t*-test showed statistically significance deviations between the observed and expected heterozygosity (*t*(18.091), 23DF, *P* = 0.000). At the infrapopulation level all the *F*_*IS*_ are positive. Fourteen on 24 values are significantly different from zero.

**Table 9:** Expected and observed heterozygosity with mean number of allele count and F_*IS*_ for each infrapopulation

Patient	Sample size	Loci typed	*H*_*E*_	*H*_*E*_ SD	*H*_*O*_	*H*_*O*_ SD	No. Alleles	No Alleles SD	*F*_*IS*_	95 % CI
K1	10	11	0.6824	0.0867	0.4601	0.0554	5.00	1.90	**0.34494**	(0.05579–0.43622)
K2	10	11	0.6290	0.0938	0.3938	0.0515	5.55	3.14	**0.38889**	(0.20615–0.43191)
K3	8	11	0.6753	0.0913	0.4074	0.0583	4.82	1.83	**0.41958**	(0.11086–0.49138)
K9	10	11	0.6603	0.0560	0.4701	0.0489	5.18	2.44	**0.29971**	(0.15599–0.33207)
K12	10	11	0.6559	0.0794	0.4735	0.0499	6.64	3.47	**0.29048**	(0.11189–0.34722)
K13	10	11	0.6984	0.0714	0.5205	0.0518	5.91	2.77	**0.26863**	(0.05067–0.30071)
K14	10	11	0.6808	0.0716	0.4436	0.0521	5.45	1.92	**0.36387**	(0.16972–0.41061)
K15	10	11	0.7057	0.0487	0.3695	0.0594	4.36	1.80	**0.49892**	(0.23853–0.53854)
SE1	9	11	0.6557	0.0526	0.4707	0.0541	4.45	1.75	**0.34882**	(0.09111–0.42230)
SE12	29	11	0.7309	0.0372	0.5414	0.0295	7.18	3.95	**0.26324**	(0.17658–0.32751)
SE14	29	11	0.7209	0.0335	0.5562	0.0293	7.27	3.66	**0.23196**	(0.13622–0.30570)
SE16	5	11	0.7165	0.0365	0.5167	0.0762	3.55	0.93	0.30460	(–0.03072–0.38731)
W21	5	11	0.5919	0.0977	0.4318	0.0755	3.27	1.85	0.28750	(−0.13514–0.32817)
W22	6	11	0.7120	0.0777	0.4667	0.0649	4.36	1.63	0.37143	(−0.01250–0.46882)
W38	6	11	0.7057	0.0743	0.5909	0.0630	4.91	2.17	0.17635	(−0.19171–0.22309)
W47	6	11	0.7061	0.0795	0.5758	0.0623	4.73	1.74	0.20000	(−0.10236–0.21839)
W51	6	11	0.6967	0.0756	0.4364	0.0657	4.00	1.73	**0.40496**	(0.09867–0.40496)
W55	5	11	0.6683	0.0697	0.4455	0.0703	3.82	1.33	0.36087	(−0.05263–0.38804)
W62	7	11	0.7161	0.0754	0.4680	0.0650	4.45	1.63	**0.36995**	(0.04770–0.39774)
W88	6	11	0.6035	0.0960	0.4273	0.0639	3.82	1.94	0.31884	(−0.14187–0.44037)
W89	8	11	0.6612	0.0799	0.4908	0.0593	4.55	2.50	0.27769	(−0.00187–0.39571)
Z3	5	11	0.6437	0.0804	0.3485	0.0674	3.64	1.29	0.49727	(−0.21429–0.56041)
Z4	32	11	0.6530	0.0638	0.4113	0.0283	7.55	4.01	**0.37517**	(0.26578–0.46044)
Z5	5	11	0.6362	0.0821	0.2848	0.0752	3.09	1.87	0.59872	(−0.36842–0.59872)

Bold F_*IS*_ shows a posetive 95 % CI correlation

Colony analysis in Kemissie revealed an estimated total of 37 genetically unique adult worm pairs, representing 88 % of the pairs (Table 10). Twenty six pairs are shared among the eight patients sampled in Kemissie. Similarly, there were an estimated total of 36 genetically unique adult worm pairs, with a mean of 73 %, and 19 genetically similar worm pairs common to the four individuals in Sille-Elgo. In Wondo Genet there were an estimated total of 32 genetically unique adult worm pairs, with a mean of 92 %, and genetically similar worm pairs common to the nine individuals. In Ziway, a total of 24 genetically unique adult worm pairs with a mean of 66 % were observed and there were four genetically similar worm pairs common to the three individuals.

**Table 10:** Estimated sibship of *Schistosoma mansoni* in a single human host

Kemissie	Sample size	Estimated genetically unique adult worm pairs within hosts	Unique worm pairs	Shared pairs among patients
K1	10	9 (90 %)	37	26
K2	10	10(100 %)		
K3	8	7(88 %)		
K9	10	8(80 %)		
K12	10	9(90 %)		
K13	10	9(90 %)		
K14	10	8(80 %)		
K15	10	9(90 %)		
Mean		88 %		
Sille-Elgo				
SE1	9	7(78 %)	36	19
SE12	29	23(79 %)		
SE14	29	22(76 %)		
SE16	5	3(60 %)		
Mean		73 %		
Wondo Genet				
W21	5	5(100 %)	32	15
W22	6	6(100 %)		
W38	6	6(100 %)		
W47	6	5(83 %)		
W51	6	5(83 %)		
W55	5	5(100 %)		
W62	7	7(100 %)		
W88	6	5(83 %)		
W89	8	7(88 %)		
Mean		92 %		
Ziway				
Z3	5	4(80 %)	24	4
Z4	32	19(59 %)		
Z5	5	3(60 %)		
Mean		66 %		

## Discussion

In the current study high level of *S. mansoni* genetic polymorphism was observed as evidenced by a large number of alleles detected in each population and infrapopulation. Variation in the number of alleles counted from a single host at the 11 loci level suggests that the degree of heterozygosity is highly variable among the study subjects. In a Kenyan study a relatively higher value, compared to the current study, was reported [5]. In a comparison study of infrapopulations genetic diversity between two villages in Brazil, Thiele et al. [7] reported much higher allele count than the current study. This high genetic diversity of Ethiopian *S. mansoni* isolates can also be attributed to coinfections by multiple genotypes from genetically different cercariae. Schistosome genetic diversity within molluscan host populations has been characterized in previous studies [27]. These previous studies showed that the biology of the schistosomes is such that dispersal of the parasite is dependent on the host dispersal and the dissemination of the free larval stages (miracidium and cercariae).

The large geographical distance separating the four study sites is likely to limit contacts between populations, thus promoting schistosome population differentiation among them. Allelic richness scored was higher for Kemissie followed by Wondo Genet, Ziway and Sille-Elgo. The nonparametric Friedman test showed that there is no significant difference in allelic richness among the four populations. However, Wilcoxon rank test indicated that Sille-Elgo had the lowest allelic richness, showing low genetic variation of *S. mansoni* isolates of Sille-Elgo compared to the others. Similar finding was reported in a Kenyan study [5]. It is interesting to notice that the gradient in genetic diversity (higher for Kemissie followed by Wondo Genet, and Sille-Elgo) is the same as the gradient we previously observed for prevalence and intensities of infection [1]. However, because only three populations were sampled we cannot statistically validate a link between parasite genetic diversity and parasite virulence.

In the current study there was statistically significant difference between the expected and observed heterozygosities showing significant departure from Hardy–Weinberg equilibrium. The *FIS* value for all loci in the four populations is significantly greater than zero, indicating heterozygote deficiency. However, Sille-Elgo and Ziway had less than zero value at the SMDA28 and SMBR10 loci, respectively, indicating heterozygote excess. A study in Brazil reported a bit similar result to our finding [28]. A study by Agola et al. [5] showed no statistically significant difference between expected and observed heterozygosity. In a recent study performed in Senegalese population, similar positive *Fis* values has been measured, in both population (i.e. village) and infrapopulation (i.e. human) levels [6]. The authors propose that positive *Fis* values at infrapopulation level may be a consequence of sib transmission (i.e. person visiting the same transmission site frequently). This sib transmission is particularly true for children compared to adults [6]. Because our sampling includes children younger than 14 years old a similar sib-transmission explanation agrees with *Fis* values we observed. In context of strong sib transmission a significant genetic structure would be expected as we have observed in our study. In Van den Broeck et al. [6] study the authors did not observed spatial structure and proposed that this is a consequence of high host mobility. We could thus hypothesis that the mobility of host is reduced in the present study compared to previous study in Senegal.

All the methods we used in this study (*Fst* calculation, PCA or Bayesian approach) show a clear and significant genetic differentiation, supporting distinctions among the four populations, thus implying that there is restricted gene flow among the schistosomes under study [29]. In contrast to this finding, a study in central Kenya showed that the PCA lacked clear geographical patterns suggesting the absence of strong substructure within the *S. mansoni* population [5]. However, in the current study the genetic structuration of *S. mansoni* population was not associated with isolation by distance. Guadeloupe island [30], Brazil [7], and Kenya [5, 20] had shown that the genetic structuration of *S. mansoni* population was not associated with isolation by distance which in agreement with our finding. As described by Agola et al. [5] the probable reason for the absence of association between genetic diversity and isolation by distance in this study could be due to a combination of factors that include restricted gene flow between populations since the areas are located in geographically distant sites, sib-transmission, local adaptations and systematic variations in environmental conditions. Another explanation may be the low number of studied sites does not allow showing significant statistical link between genetic and geographic distances.

The estimation of genetically unique adult worm pairs within a single host proves the occurrence of infections with genetically different *S. mansoni* strains within a single host. An investigation on the genotypic composition of *S. mansoni* for its adult stages within the definitive host (the wild rat, *Rattus rattus*) and for the larval stages within the intermediate host (the snail, *Biomphalaria glabrata*) both collected at the same transmission site was conducted by Theron and colleagues [31]. The result showed that intramolluscan larval infrapopulations were characterized by a low infection rate (0 · 6 % on average) and low intra-host genetic diversity (1 · 1 genotype on average per infected snail), while adult infrapopulations within rats showed a high infection rate (94 %) and a substantial intra-host genetic diversity (34 genotypes on average) linked to high intensities (160 worms per host on average). Analysis of the genetic data allowed them the identification of various ecological, behavioral and immunological factors which are likely to enhance transmission of multiple parasite genotypes towards the vertebrate hosts. This identification of infection of both the intermediate and definitive hosts with genetically different *S. mansoni* strains is in favor of the current finding where multiplicity of infection occurred in human hosts based on sibship determination. Colony analysis in Kemissie revealed an estimated total of 37 genetically unique adult worm pairs, with a mean of 88 % and 26 shared worm pairs among eight individuals. Similarly, there were an estimated total of 36 genetically unique adult worm pairs, with a mean of 73 %, and 19 worm pairs shared among the four individuals in Sille-Elgo. In Wondo Genet there were an estimated total of 32 genetically unique adult worm pairs, with a mean of 92 %, and 15 worm pairs shared among the nine individuals. In Ziway, a total of 24 genetically unique adult worm pairs with a mean of 66 % were observed and there were 4 worm pairs shared among the three individuals. This result showed high levels of infection of single host with genetically different multiple strains of *S. mansoni* isolates.

## Conclusion

The study revealed that the schistosome isolates from the four study sites in Ethiopia have highly polymorphic allele counts and are genetically structured. These results are quite different from previous studies demonstrating that it is difficult to generalize schistosome transmission patterns because epidemiological situation tends to vary. These are important factors to be considered in relation with morbidity, drug resistance or vaccine development. The presence of a rich snail fauna in East Africa (about half a dozen species of *Biomphalaria* and *Bulinus*), including Ethiopia could be the source of high genetic differentiation and structuration. Therefore, further investigation of *S. mansoni* isolates in different localities and also its interaction with host snails is recommended to determine the phylogeography of *S. mansoni* isolates of Ethiopia. The human behavior effect in *S. mansoni* population structuration and if there are other reservoir hosts that can have effect on the *S. mansoni* population structure should be investigated.
